# Protective Effects of *Lacticaseibacillus paracasei* 63 on DSS-Induced Colitis in Mice

**DOI:** 10.3390/foods15111932

**Published:** 2026-05-29

**Authors:** Yunchao Wa, Min Liu, Huandong Yang, Nan Ling, Huafang Xu, Yujun Huang, Dawei Chen, Chenchen Zhang, Ruixia Gu

**Affiliations:** 1 The School of Food Science and Engineering, Yangzhou University, Yangzhou 225000, China; 008213@yzu.edu.cn (Y.W.); 15163253370@163.com (M.L.); yjhuang@yzu.edu.cn (Y.H.); chendawei0816@163.com (D.C.); cczhang@yzu.edu.cn (C.Z.); 2 Key Laboratory of Probiotics and Dairy Processing in Provincial Universities, Yangzhou University, Yangzhou 225000, China; 3 Jiangsu Weigang Dairy Research Institute Co., Ltd., Nanjing 211106, China; 807509@wgdairy.com.cn (H.Y.); lann81@163.com (N.L.); xuhf@wgdairy.com.cn (H.X.)

**Keywords:** ulcerative colitis, *Lacticaseibacillus paracasei*, inflammatory response, intestinal barrier function, gut microbiota

## Abstract

Ulcerative colitis (UC) is a chronic inflammatory disease of the colon characterized by mucosal damage and immune dysregulation. This study aimed to evaluate the protective effects of *Lacticaseibacillus paracasei* 63 on ulcerative colitis. A dextran sulfate sodium (DSS)-induced male ICR mouse model of colitis was employed to assess the effects of oral administration of *L. paracasei* 63 on intestinal inflammation, barrier integrity, gut microbiota composition, short-chain fatty acid (SCFA) production, systemic inflammatory markers, and primary immune organ indices. Results showed that *L. paracasei* 63 significantly alleviated colitis and improved intestinal barrier function, accompanied by reduced TLR4 expression, decreased NF-κB p65 phosphorylation, and decreased MLCK-related markers. In addition, *L. paracasei* 63 administration modulated gut microbiota composition and increased SCFA levels, particularly acetate. These local intestinal changes were accompanied by reduced systemic inflammation, as evidenced by decreased serum IL-1β and TNF-α levels, along with an increased thymus index. Overall, *L. paracasei* 63 exerted significant ameliorative effects on DSS-induced ulcerative colitis, and these effects were associated with improvements in intestinal barrier integrity, inflammatory status, gut microbiota composition, SCFA production, and systemic immune-related indicators.

## 1. Introduction

Ulcerative colitis (UC) is a chronic, relapsing inflammatory bowel disease characterized by persistent inflammation and structural damage of the colonic mucosa, severely impairing patients’ quality of life [1]. In addition to typical intestinal manifestations such as abdominal pain, diarrhea, and bloody stools, UC is frequently accompanied by systemic inflammatory responses affecting multiple organs and physiological systems, leading to complications including weight loss, anemia, arthritis, and metabolic disorders [2]. Thus, UC is increasingly recognized as a disease associated with systemic immune dysregulation rather than a purely localized intestinal disorder [3].

Recent advances have highlighted the role of gut microbiota and their metabolites in modulating host immune responses [4,5]. Probiotics have been widely reported to alleviate intestinal inflammation in UC, primarily through regulating microbial composition, enhancing barrier function, and modulating local immune responses [6,7]. Meanwhile, microbial-derived metabolites, particularly short-chain fatty acids (SCFAs), are implicated in immune regulation beyond the intestinal environment [8]. These findings suggest that intestinal homeostasis may be linked to systemic immune status, although the extent to which such intestinal improvements translate into systemic immunological changes remains unclear.

*L. paracasei* is a widely studied probiotic species with documented anti-inflammatory and immunomodulatory properties [9,10]. Several *L. paracasei* strains have been reported to alleviate DSS-induced colitis in animal models. However, probiotic effects are highly strain-dependent, and findings obtained from one strain cannot be directly generalized to another strain within the same species. Therefore, strain-specific evaluation remains necessary to determine the therapeutic potential of individual probiotic candidates. In our preliminary screening using an LPS-induced Caco-2 and THP-1 co-culture model, *L. paracasei* 63 exhibited notable anti-inflammatory activity, characterized by reduced pro-inflammatory cytokine expression and enhanced tight junction protein expression. These findings suggest its potential protective effects on intestinal inflammation and epithelial barrier integrity. However, the in vivo efficacy of *L. paracasei* 63 in alleviating UC has not been systematically evaluated.

In this study, we evaluated the ameliorative effects of *L. paracasei* 63 on colitis and associated systemic inflammation using a DSS-induced murine model. Specifically, we assessed its impact on intestinal inflammation, barrier integrity, gut microbiota composition, and systemic inflammatory responses. In addition, we explored the potential association between *L. paracasei* 63 intervention and thymus index as a preliminary indicator of systemic immune relevance. This study aims to provide strain-specific evidence for the protective effects of *L. paracasei* 63 against DSS-induced colitis and to offer preliminary insights into the possible link between intestinal homeostasis and systemic immune status.

## 2. Materials and Methods

### 2.1. Strains and Culture

The bacterial species *L*. *paracasei* 63 (CGMCC No. 31768) and *Bifidobacterium animalis* subsp. *Lactis* BB12 (*B. animalis* BB12) were used in this study. Activated cultures of *L*. *paracasei* 63 and *B. animalis* BB12 were inoculated into de Man, Rogosa, and Sharpe (MRS) and modified MRS (with added cysteine and mupirocin lithium salt) medium (Haibo Biotechnology Co., Ltd., Qingdao, China), followed by incubation at 37 °C for 24 h.

### 2.2. Animal Experimental Design and Sample Collection

Male ICR mice (*n* = 40; age: five weeks old; weight: 25 ± 1.0 g) were purchased from the Institute of Comparative Medicine of Yangzhou University (Jiangsu, China). The mice were individually housed in stainless steel cages in a pathogen-free room at 22 ± 2 °C and 55 ± 5% relative humidity under a 12 h/12 h light-dark cycle. All animal procedures complied with the Guide for the Care and Use of Laboratory Animals and were approved by the Animal Care Committee of the Centers for Disease Control and Prevention (Approval No. 202503176, Jiangsu Province, China).

All mice were acclimated for one week, and each mouse was randomly assigned to one of four groups (*n* = 10 mice per group): Normal control (NC), DSS model group (M), *L. paracasei* 63 group (L.pc 63), and *B. animalis* BB12 group (BB12). The experiment lasted 21 days; during the first seven days, groups M, L.pc 63, and BB12 were administered 3% DSS in drinking water.

**Figure 1:**
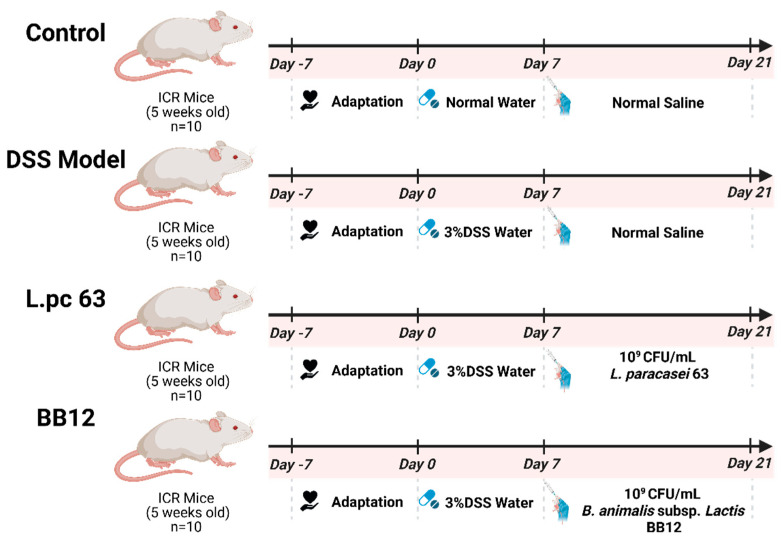
Schematic illustration of the experimental design.

From days 8 to 21, the control group and the M group were administered physiological saline (0.9% NaCl) every day, whereas the L.pc 63 group was intragastrically administered a 200 μL suspension of *L. paracasei* 63 (10^9^ CFU·mL^−1^). The BB12 group was intragastrically administered a 200 μL suspension of *B. animalis* BB12 (10^9^ CFU·mL^−1^), once a day for 14 consecutive days (Figure 1). During this period, mice had free access to drinking water and food, which were replenished regularly (Figure 1). Throughout the experiment, the body weight of the mice was recorded daily, and feces were collected every day to analyze fecal characteristics and occult blood. After the mice were euthanized, their colonic contents were immediately collected for subsequent 16S rRNA gene sequencing. Tissues from the colon were collected and rapidly frozen in liquid nitrogen for subsequent analyses. After removing and weighing the spleen and thymus, their respective indices were calculated using the following formulae: Spleen index (mg/g) = spleen weight (mg)/body weight (g); Thymus index (mg/g) = thymus weight (mg)/body weight (g).

### 2.3. Histopathology

Histopathological analysis of colon tissues was performed with reference to a previously described method with minor modifications [11]. Briefly, distal colon samples were fixed in 4% paraformaldehyde for 48 h, followed by routine paraffin embedding and sectioning at a thickness of 5 μm. The sections were then subjected to hematoxylin and eosin (H&E) staining to assess general tissue morphology and Alcian Blue–Periodic Acid-Schiff (AB-PAS) staining to observe mucus-producing goblet cells.

Stained sections were examined using a light microscope (Nikon E100, Nikon, Tokyo, Japan). For H&E-stained sections, mucosal integrity, epithelial structure, submucosal edema, inflammatory cell infiltration, and crypt architecture were descriptively evaluated. For AB-PAS-stained sections, mucus-containing goblet cells and crypt-related morphological features were observed. In this study, H&E and AB-PAS staining were used to provide representative morphological evidence of colonic tissue injury and goblet cell-related changes, rather than for quantitative histopathological scoring or goblet cell counting.

### 2.4. Enzyme-Linked Immunosorbent Assay (ELISA)

To conduct ELISA, blood samples of mice were collected in vacuum tubes with red lids. After the samples were left undisturbed for 2 h at 37 °C, they were incubated overnight at 4 °C. Next, the serum was allowed to precipitate, after which the samples were centrifuged at 3000 rpm for 10 min at 4 °C to obtain serum, which was sub-packed and stored at −80 °C for ELISA. The intestinal permeability indicators, including serum diamine oxidase (DAO), D-lactate (D-LA), and lipopolysaccharide (LPS), along with inflammatory indicators, including IL-1β, IL-6, and TNFα, were estimated by ELISA (Elabscience, Bethesda, MD, USA).

### 2.5. Immunohistochemistry Examinations

Immunofluorescence staining of tight junction proteins was conducted with reference to a previously reported protocol with minor modifications [6]. In brief, colon tissue sections were incubated overnight at 4 °C with primary antibodies against ZO-1, Claudin-1, or Occludin (1:200), followed by incubation with IgG secondary antibodies (1:200) at room temperature for 2 h. Cell nuclei were counterstained with DAPI (Solarbio, Beijing, China), and the sections were mounted using an anti-fading solution.

Fluorescence images were captured under a microscope (Olympus, Tokyo, Japan) at 200× magnification. The staining was used to compare the localization and relative expression patterns of ZO-1, Claudin-1, and Occludin in colon tissues.

### 2.6. Quantitative Real-Time Polymerase Chain Reaction (qRT-PCR)

We isolated total RNA from colon tissues using a UNlQ-10 Column Trizol Total RNA Isolation Kit (Sangon Biotech Co., Ltd., Shanghai, China) following the manufacturer’s protocol. The RNA concentration and purity were measured using a NanoPhotometer spectrophotometer (Implen, Munich, Germany). The primers used for the reference gene (β-actin) and target genes were designed using the online software Primer 5.0 and are shown in Table 1. Then, we performed quantitative real-time PCR using an ABI Prism 7300 Detection System (Applied Biosystems, Foster City, CA, USA). All reactions were performed in duplicate using a suitable kit purchased from TaKaRa Biotechnology Company (San Jose, CA, USA). The relative mRNA levels of the genes were determined using the 2^−ΔΔCt^ method, with β-actin as the reference gene. For each target gene, ΔCt values were calculated relative to β-actin, and ΔΔCt values were calculated using the NC group as the calibrator. Fold changes were then calculated using the 2^−ΔΔCt^ method.

### 2.7. Quantification of the SCFAs

Short-chain fatty acids (SCFAs) were quantified following the method described in a previous study [5]. Fecal samples for SCFA analysis were collected from mice on day 20, immediately frozen, and stored at −80 °C until analysis. Briefly, 0.50 g of feces from each mouse was suspended in 900 μL of 0.5% phosphate buffer and centrifuged at 14,000× *g* for 10 min. Then, 800 μL of the supernatant was collected and extracted with an equal volume of ethyl acetate, followed by centrifugation at 14,000× *g* for 10 min. Next, a 600 μL aliquot of the organic phase was filtered through a 0.45 μm membrane. The internal standard, 500 μM 4-methylpentanoic acid, was then added to the filtrate, thoroughly mixed, and transferred to a vial for further analysis. The samples were analyzed by GC-MS using an Agilent DB-WAX capillary column (Agilent Technologies, Santa Clara, CA, USA). Chromatographic peak areas and retention times were extracted using MSD ChemStation software, version E.02.02. SCFA concentrations were calculated based on standard curves.

### 2.8. 16S rRNA Gene Sequencing

We conducted 16S rRNA gene sequencing using methodologies described in another study [12]. To investigate the composition of the gut microbiome in mice, colon contents were collected from mice on day 22. Then, 16S rRNA gene sequencing was used to detect intestinal flora in colon contents by Shanghai Majorbio Bio-pharm Technology Co., Ltd. (Shanghai, China). The primers 338F (5-ACTCCTACGGGAGGCAGCAG-3) and 806R (5-GGACTACHVGGGTWTCTAAT-3) were selected to amplify this region. First, the whole genome DNA was extracted and amplified; equimolar amounts of PCR products were quantified and pooled. Finally, the completed library was sequenced using an Illumina Nextseq2000 platform (Illumina, San Diego, CA, USA).

### 2.9. Western Blotting Analysis

Western blot analysis was conducted according to a previously reported protocol with minor modifications [9]. Colon tissue samples from three independent biological replicates per group were used for protein extraction, and protein concentrations were measured with a BCA protein assay kit (Solarbio, Beijing, China). Equal amounts of protein were resolved by SDS-PAGE, transferred to PVDF membranes (Merck Millipore, Burlington, MA, USA), blocked, and incubated with primary antibodies against MLCK, p-MLC2, MLC2, TLR4, p-P65, P65, and β-actin (1:1000, Proteintech Co., Ltd., Wuhan, China). After incubation with secondary antibodies, the protein bands were detected using an Amersham Imager 600 system (General Electric Healthcare Life Sciences, Chicago, IL, USA).

Band intensities were quantified using the AlphaEaseFC software, version 4.0 processing system. Representative blots were selected from independent biological samples and were consistent with the quantitative results. Total protein levels, including MLCK, MLC2, TLR4, and P65, were normalized to β-actin. Phosphorylated protein levels were calculated as the ratio of p-MLC2 to total MLC2 and p-P65 to total P65 after β-actin normalization.

### 2.10. Statistical Analysis

All data are presented as the mean ± SD. Statistical analyses were performed using GraphPad Prism software (version 10.0.0 for Windows, GraphPad Software, Boston, MA, USA). Comparisons among multiple groups were conducted using one-way analysis of variance (ANOVA) followed by Tukey’s post hoc multiple comparisons test. A value of *p* < 0.05 was considered statistically significant. The exact sample size (*n*) for each experiment is indicated in the corresponding figure legends.

## 3. Results

### 3.1. L. paracasei 63 Alleviated Symptoms of UC in Mice

**Figure 2:**
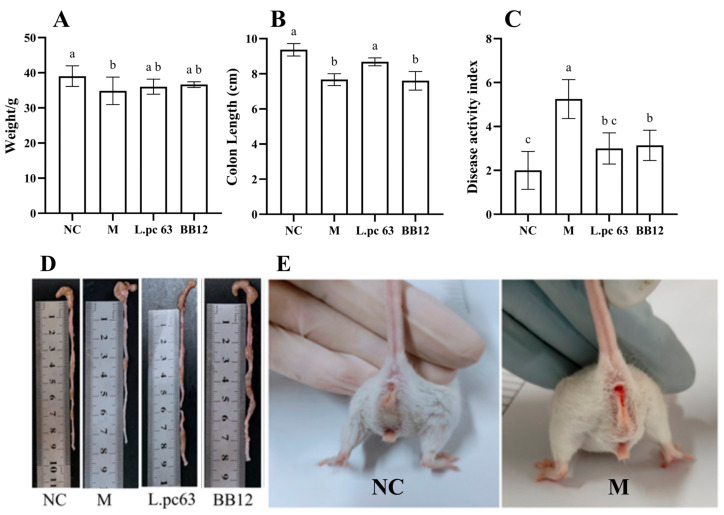
*L. paracasei* 63 alleviated symptoms of DSS-induced UC in mice, including body weight (**A**), colon length (**B**), disease activity index (**C**), representative images showing the colon lengths (**D**), representative images comparing symptoms of colitis in mice (**E**). NC, normal control group; M, DSS model group; L.pc 63, *L. paracasei* 63 group; BB12, *B. animalis* BB12 group. Different lowercase letters in the figure indicate significant differences between groups, *p* < 0.05, *n* = 10.

**Figure 3:**
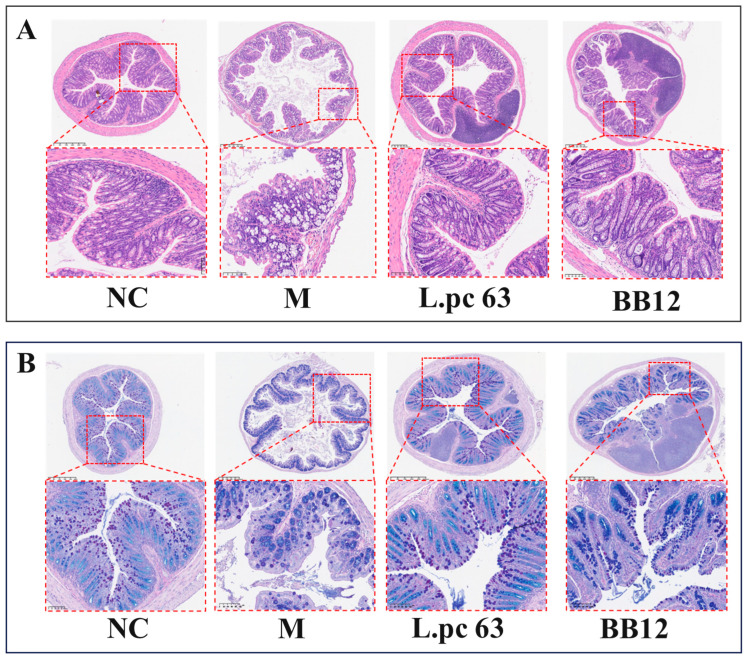
*L. paracasei* 63 alleviated the histopathological lesions of the colon and intestinal mucosa in DSS-induced UC mice. The image shows hematoxylin and eosin (H&E)-stained histological colon tissue; scale bar: 100 µm, *n* = 3 (**A**). The image shows Alcian Blue- Periodic Acid-Schiff (AB-PAS) histological colon tissue; scale bar: 200 µm, *n* = 3 (**B**). NC, normal control group; M, DSS model group; L.pc 63, *L. paracasei* 63 group; BB12, *B. animalis* BB12 group.

The mice in the M group had significantly lower body weight and shorter colon length than those in the NC group (*p* < 0.05; Figure 2A–C). Additionally, pathological alterations were substantial in the M group, as determined by H&E and AB-PAS staining, including the rupture of villi, mucus vacuolization, damage to the mucosal epithelium, loss of goblet cells, crypt destruction, and severe infiltration of inflammatory cells (Figure 3A,B). These findings indicated that DSS-induced UC in mice manifested as weight loss, colon shortening, intestinal pathology, and activation of the immune system.

The body weight of the mice in the L.pc 63 and BB12 groups was higher than that of the mice in the M group, but the difference in body weight was not significant compared to that of the mice in the NC group (*p* > 0.05; Figure 2A). The length of the colon in the L.pc 63 and BB12 groups was significantly greater than that of the M group (*p* < 0.05; Figure 2B). The disease activity index (DAI) in both groups was significantly lower than that of the M group (*p* < 0.05; Figure 2C). Colon length was significantly greater in the L.pc 63 group than in the BB12 group and was not significantly different compared to that in the NC group (*p* < 0.05; Figure 2B). Moreover, in the L.pc 63 group, colon tissue had tightly arranged cells, reduced inflammatory cell infiltrates, and normal crypt architecture approaching that of the NC group (Figure 3A,B), which indicated that interventions with *L. paracasei* 63 and *B. animalis* BB12 considerably ameliorated UC symptoms in mice.

### 3.2. L. paracasei 63 Alleviated Colonic Inflammatory Responses in Mice

**Figure 4:**
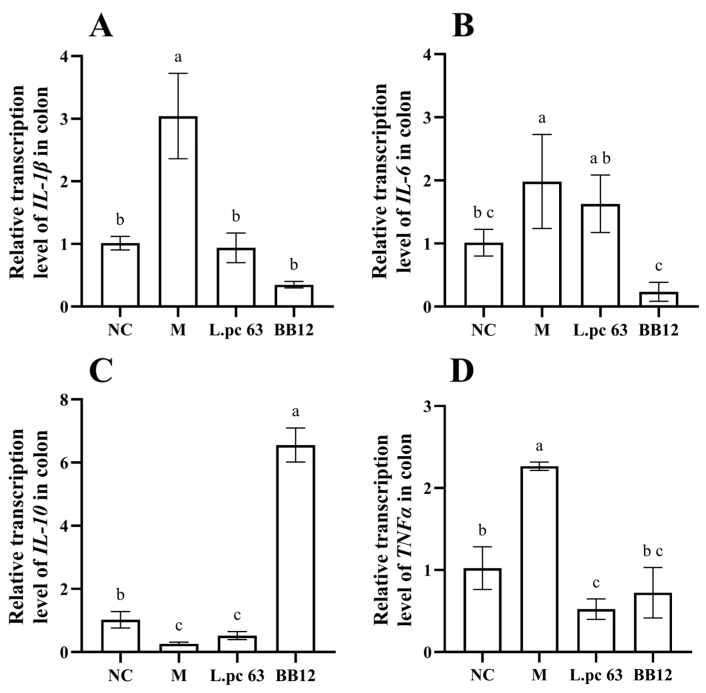
Effects of *L. paracasei* 63 on colonic inflammatory cytokine expression in DSS-induced UC mice. mRNA expression levels of IL-1β (**A**), IL-6 (**B**), IL-10 (**C**), and TNF-α (**D**). NC, normal control group; M, DSS model group; L.pc 63, *L. paracasei* 63 group; BB12, *B. animalis* BB12 group. Different lowercase letters in the figure indicate significant differences between groups (*p* < 0.05), *n* = 3.

The transcription levels of the pro-inflammatory factors *IL-1β*, *IL-6*, and *TNFα* in the intestines of mice in the M group were significantly higher than their respective levels in the NC group (*p* < 0.005, *p* < 0.05, and *p* < 0.001; Figure 4A,B,D). In contrast, the transcription level of the anti-inflammatory factor *IL-10* was significantly lower in the M group than in the NC group (*p* < 0.001; Figure 4C). This indicated that DSS stimulation triggered an inflammatory response in the colons of the mice.

The transcription level of *IL-1β* in the colon was downregulated in the L.pc 63 and BB12 groups compared to that in the M group (*p* < 0.001, Figure 4A) but not significantly different compared to that in the NC group (*p* > 0.05, Figure 4A). The transcription level of IL-6 was significantly reduced in both the L.pc 63 and BB12 groups compared with that in the M group. Compared with the NC group, the IL-6 level in the L.pc 63 group showed no significant difference, whereas that in the BB12 group remained significantly lower (*p* < 0.05; Figure 4B). The transcription level of *IL-10* was upregulated in the L.pc 63 and BB12 groups compared to that in the M group; however, the L.pc 63 group showed significant downregulation compared to the NC group, whereas the BB12 group showed significant upregulation compared to the NC group (*p* < 0.05 and *p* < 0.001; Figure 4C). The transcription level of *TNFα* was downregulated in the L.pc 63 and BB12 groups compared to that in the M group. The L.pc 63 group showed significant downregulation compared to the NC group (*p* < 0.001; Figure 4D), while the BB12 group exhibited no significant difference from the NC group (*p* > 0.05; Figure 4D). These results indicated that *L. paracasei* 63 can alleviate intestinal inflammation by downregulating the transcription of inflammatory factors.

**Figure 5:**
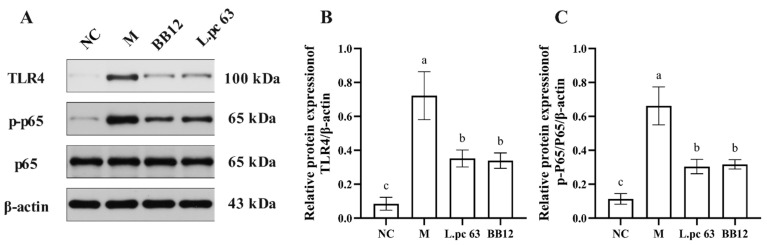
*L. paracasei* 63 modulated NF-κB activation in colonic tissue. (**A**) NF-κB, TLR4, and p65 protein levels in the colonic tissues were analyzed by Western blotting. (**B**) The expression of TLR4 was normalized to the expression of β-actin. (**C**) The phosphorylation of P65 was normalized to β-actin. NC, normal control group; M, DSS model group; L.pc 63, *L. paracasei* 63 group; BB12, *B. animalis* BB12 group. Different lowercase letters in the figure indicate significant differences between groups (*p* < 0.05), *n* = 3.

To determine the mechanism by which L.pc 63 alleviates intestinal inflammation in mice, we measured the expression levels of TLR4 protein and the phosphorylation levels of P65 protein. The levels of TLR4 and p-P65/P65 increased by 8.49-fold and 7.79-fold, respectively, in the M group compared to their levels in the NC group (*p* < 0.05; Figure 5B,C), which indicated that DSS drives the intestinal inflammatory response in the mice in the M group through overactivation of the TLR4/NF-κB signaling axis. The levels of TLR4 and p-P65/P65 in the colon of mice in the L.pc 63 and BB12 groups were significantly lower compared to their respective levels in the colon of mice in the M group (*p* < 0.05; Figure 5B,C). These results suggested that *L. paracasei* 63 may alleviate inflammatory responses in the colon by regulating the TLR4/NF-κB signaling axis and downregulating the transcription levels of genes related to the inflammatory factors in the intestine.

### 3.3. L. paracasei 63 Alleviated Systemic Inflammation in Mice

**Figure 6:**
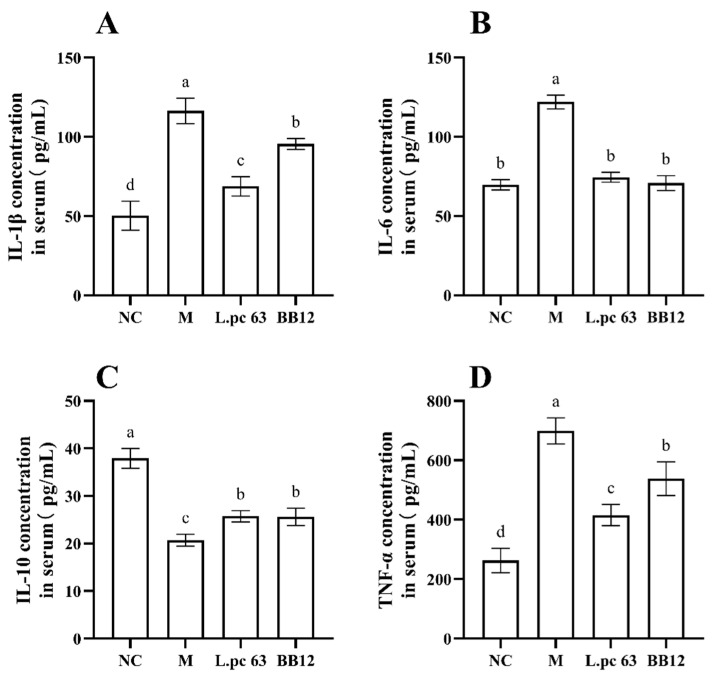
*L. paracasei* 63 can reduce serum inflammatory factor levels in DSS-induced UC mice. (**A**) IL-1β; (**B**) IL-6; (**C**) IL-10; (**D**) TNFα. NC, normal control group; M, DSS model group; L.pc 63, *L. paracasei* 63 group; BB12, *B. animalis* BB12 group. Different lowercase letters in the figure indicate significant differences between groups (*p* < 0.05), *n* = 3.

The concentrations of IL-1β, IL-6, and TNFα in the serum of mice in the M group were significantly higher than those in the serum of mice in the NC group (*p* < 0.05; Figure 6A,B,D), whereas, the transcription level of IL-10 in the M group was significantly lower than that in the NC group (*p* < 0.05; Figure 6C). This indicated that following DSS stimulation, the levels of intestinal and serum inflammatory factors in mice increased, which not only resulted in intestinal inflammation but also elicited a systemic inflammatory response. The levels of IL-1β, IL-6, and TNFα were significantly lower in the L.pc 63 group than their respective levels in the M group (*p* < 0.05; Figure 6A,B,D), and the concentration of IL-10 was significantly higher in the L.pc 63 group than that in the M group (*p* < 0.05; Figure 6C), suggesting that *L. paracasei* 63 significantly reduced systemic inflammatory responses in these mice. The levels of IL-1β and TNFα in serum in the L.pc 63 group were significantly lower than those in the BB12 group (*p* < 0.05; Figure 6A,D), which indicated that *L. paracasei* 63 can more effectively alleviate systemic inflammation in mice.

**Figure 7:**
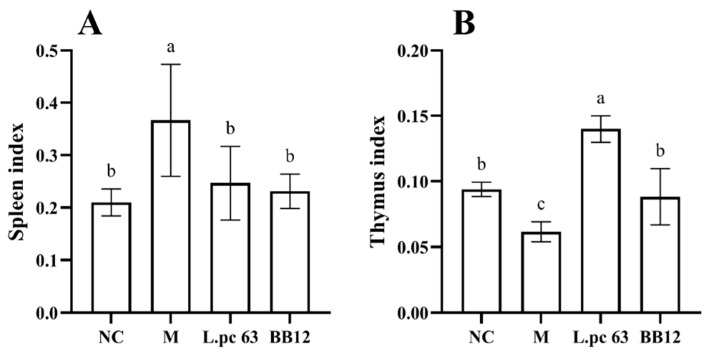
*L. paracasei* 63 affects the organ index in DSS-induced UC mice. (**A**) Spleen index; (**B**) thymus index. NC, normal control group; M, DSS model group; L.pc 63, *L. paracasei* 63 group; BB12, *B. animalis* BB12 group. Different lowercase letters in the figure indicate significant differences between groups (*p* < 0.05), *n* = 6.

The spleen index of mice in the M group was significantly increased, whereas their thymus index was significantly decreased (Figure 7A,B; *p* < 0.05), which further indicated that DSS-induced UC not only triggered intestinal inflammation but also elicited a systemic inflammatory response, leading to atrophy of the thymus (an immune organ). The spleen index of mice was significantly lower in the L.pc 63 and BB12 groups than in the M group; however, the spleen index was not significantly different between these two groups and the NC group (*p* < 0.05; Figure 7A). The thymus index in the L.pc 63 group was significantly higher than in the other groups (*p* < 0.05; Figure 7B), which indicated that *L. paracasei* 63 intervention promoted the development of the thymus in mice. This finding may be associated with the reduced levels of inflammatory factors in the serum of mice.

### 3.4. L. paracasei 63 Improved the Permeability of the Intestinal Barrier in UC Mice

**Figure 8:**
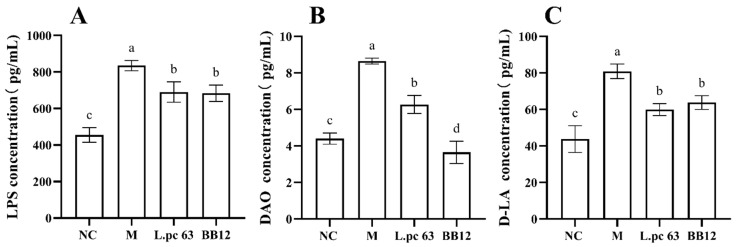
*L. paracasei* 63 improved intestinal barrier function in UC mice. Serum levels of lipopolysaccharide (LPS) (**A**), diamine oxidase (DAO) (**B**), and D-lactate (D-LA) (**C**). NC, normal control group; M, DSS model group; L.pc 63, *L. paracasei* 63 group; BB12, *B. animalis* BB12 group. Different lowercase letters in the figure indicate significant differences between groups (*p* < 0.05), *n* = 6.

The serum concentrations of LPS, D-LA, and DAO were significantly higher in the M group than in the NC group (*p* < 0.05; Figure 8A–C), which indicated DSS-induced impairment of the intestinal mechanical barrier with greater permeability. In contrast, the serum LPS, D-LA, and DAO levels were significantly lower in the L.pc 63 and BB12 groups than in the M group (Figure 8A–C; *p* < 0.05), which suggested that *L. paracasei* 63 can repair DSS-induced damage to the intestinal mechanical barrier and reduce the permeability of the barrier.

**Figure 9:**
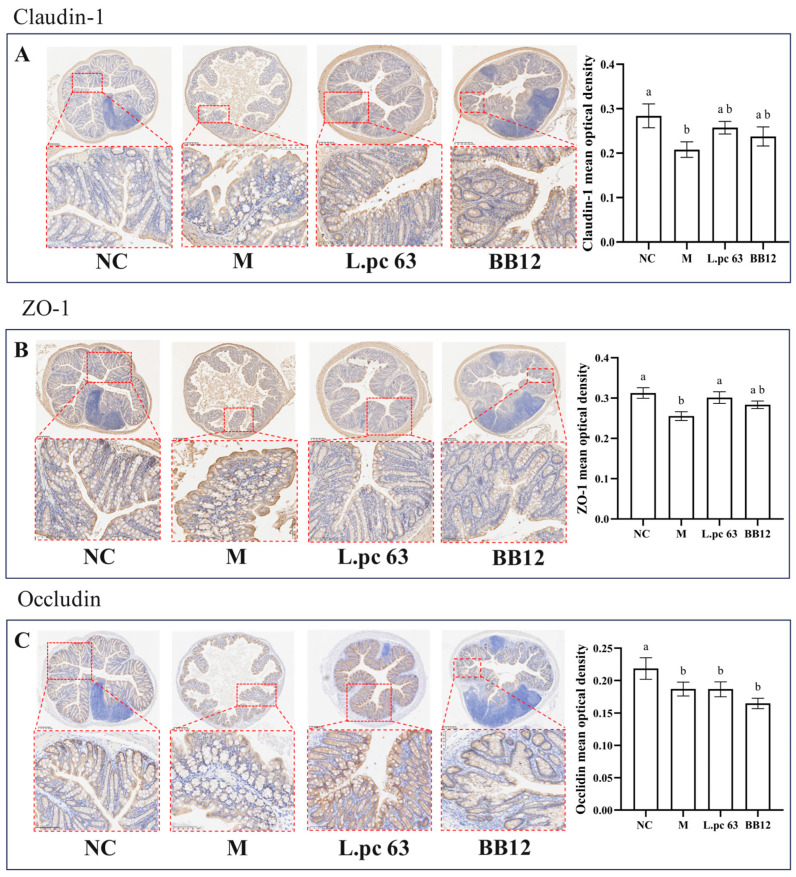
*L. paracasei 63* improved intestinal barrier tight junction integrity in UC mice. Representative immunohistochemical images and quantitative analysis of claudin-1 (**A**), ZO-1 (**B**), and occludin (**C**) expression in colon tissues. NC, normal control group; M, DSS model group; L.pc 63, *L. paracasei* 63 group; BB12, *B. animalis* BB12 group. Different lowercase letters in the figure indicate significant differences between groups (*p* < 0.05), *n* = 3. The scale bar is 300 µm.

We also measured the expression of tight junction-related genes and proteins. The levels of ZO-1, Claudin-1, and Occludin proteins were significantly lower in the M group than in the NC group (*p* < 0.05; Figure 9A–C), which indicated that stimulation by DSS suppressed the expression of tight junction-related proteins in intestinal epithelial cells, concomitantly causing intestinal injury and impairing the self-repair ability of the gut barrier. The Claudin-1 and ZO-1 protein levels in the colon were significantly higher in the L.pc 63 and BB12 groups than in the M group (*p* < 0.05; Figure 9A–C); however, they were not significantly different between these groups and the NC group. The level of ZO-1 was significantly higher in the L.pc 63 group than in the M group (*p* < 0.05; Figure 9A–C). This suggests that *L. paracasei* 63 may repair the intestinal barrier by promoting the expression of tight junction proteins in the colon.

**Figure 10:**
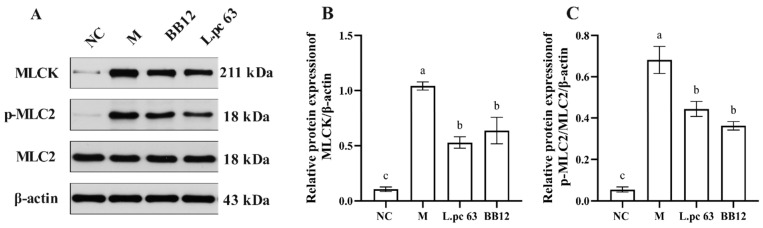
*L. paracasei* 63 modulated the activation of MLCK in colonic tissue. (**A**) MLCK, p-MLC2, and MLC2 protein levels in the colonic tissues were analyzed by Western blotting. (**B**) The expression of MLCK was normalized to β-actin. (**C**) The phosphorylation of MLC2 was normalized to β-actin. NC, normal control group; M, DSS model group; L.pc 63, *L. paracasei* 63 group; BB12, *B. animalis* BB12 group. Different lowercase letters in the figure indicate significant differences between groups (*p* < 0.05), *n* = 3.

To gain insights into the mechanisms underlying intestinal barrier repair, we measured the phosphorylation levels of the MLCK protein and its downstream effector p-MLC2 in colon tissues. The expression of the MLCK protein was increased by 9.91-fold in the M group compared to that in the NC group (*p* < 0.05; Figure 10B), and the p-MLC2/MLC2 ratio was concomitantly increased by 12.39-fold (*p* < 0.05; Figure 10C). This indicated that DSS-induced inflammation activates the MLCK signaling pathway, thereby increasing the phosphorylation of MLC2, which in turn damages tight junction proteins and increases the permeability of the intestinal barrier. In contrast, the levels of MLCK and p-MLC2/MLC2 were significantly lower in the L.pc 63 and BB12 groups than in the M group (Figure 10A,B), suggesting that *L. paracasei* 63 can restore the intestinal barrier by modulating and inhibiting the MLCK signaling pathway and the phosphorylation of MLC2.

### 3.5. L. paracasei 63 Ameliorated Gut Microbiota Dysbiosis in UC Mice

**Figure 11:**
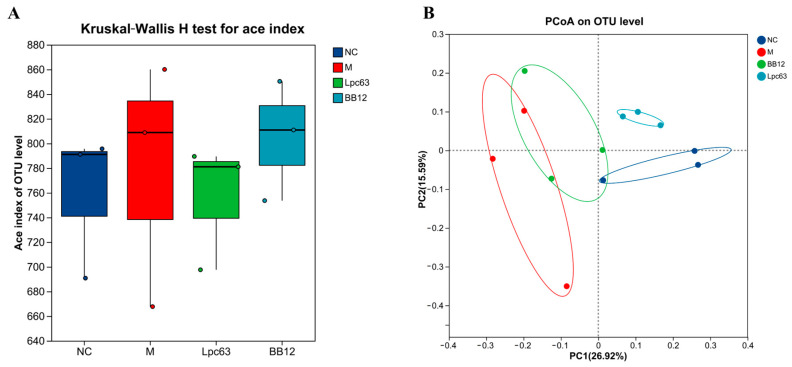
The effects of *L. paracasei* 63 on the gut microbiome composition in UC mice are presented. (**A**) Ace index. (**B**) PCoA was performed to determine the dissimilarities in microbial composition of each group. NC, normal control group; M, DSS model group; L.pc 63, *L. paracasei* 63 group; BB12, *B. animalis* BB12 group. *n* = 3.

The ACE index of OTU levels in the gut across all groups showed no significant differences (Figure 11A, *p* > 0.05), indicating that DSS-induced colitis did not reduce the overall abundance of intestinal bacteria in mice. However, the dispersion of the gut microbiota was greater in the M group than in the other groups. In the β-diversity analysis at the genus level, conducted via PCoA, the colonic microbiota structure of the M group differed considerably from the NC and L.pc 63 groups, while the NC and L.pc 63 groups exhibited more similar microbial structures (Figure 11B). These results suggested that DSS modeling significantly altered the gut microbiota composition, and *L. paracasei* 63 intervention brought the microbial structure of treated mice closer to that of the NC group.

**Figure 12:**
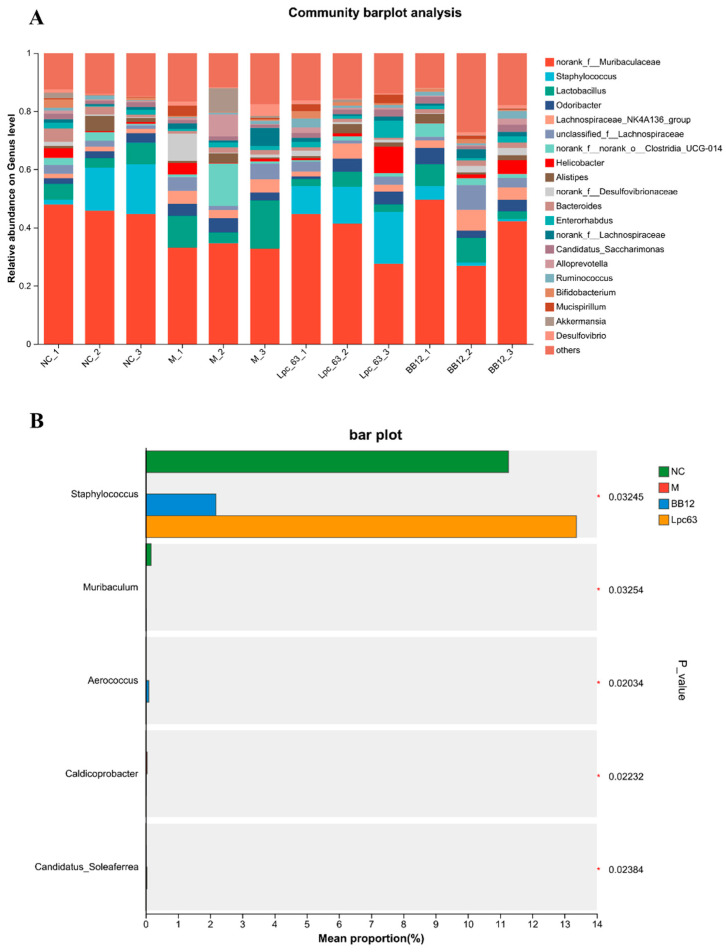
The effects of *L. paracasei* 63 on the gut microbiome compositions in DSS-induced colitis mice are presented. (**A**) The dissimilarities in microbial composition of each group at the genus level are presented. (**B**) A significance test was conducted for differences in microbial composition of each group at the genus level. NC, normal control group; M, DSS model group; L.pc 63, *L. paracasei* 63 group; BB12, *B. animalis* BB12 group. *n* = 3, * *p* < 0.05.

In the abundance analysis at the genus level and differential species analysis, compared to the mice in the NC group, those in the M group showed a significant decrease in the relative abundances of *Staphylococcus*, *Muribaculum*, *Aerococcus*, and *Candidatus_Soleaferrea*, with a significant increase in *Caldicoprobacter* (Figure 12B). The abundances of *Staphylococcus*, *Muribaculum*, *Aerococcus*, and *Candidatus_Soleaferrea* were significantly higher in the L.pc 63 and BB12 groups than in the M group, while *Caldicoprobacter* showed no significant difference between the BB12 and M groups (Figure 12B). These results suggested that *L. paracasei* 63 interventions partly restored the colonic microbial community structure in these mice.

### 3.6. L. paracasei 63 Promoted the Secretion of SCFAs in the Colon of UC Mice

**Figure 13:**
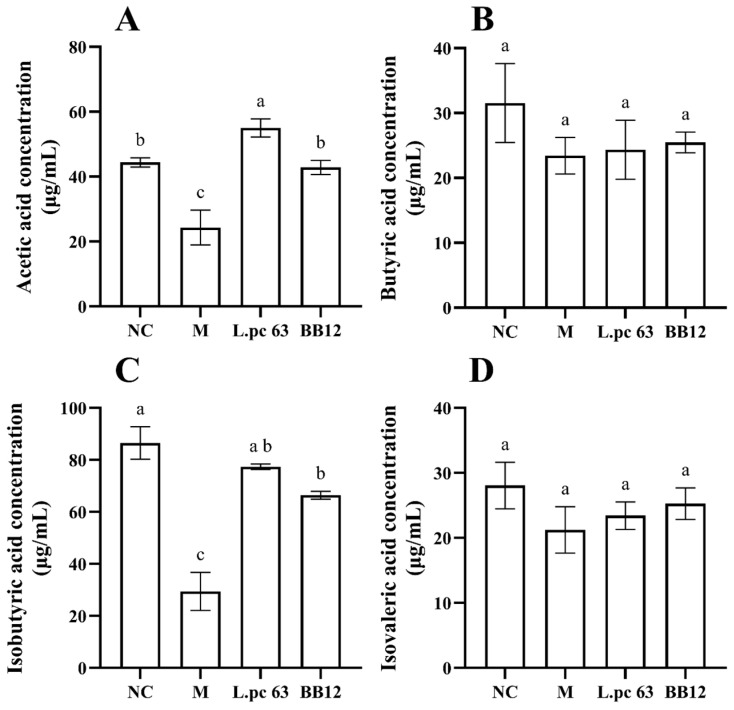
*L. paracasei* 63 promotes secretion of SCFAs in the colon of mice with UC (**A**) Acetic acid, (**B**) butyric acid, (**C**) isobutyric acid, and (**D**) isovaleric acid. NC, normal control group; M, DSS model group; L.pc 63, *L. paracasei* 63 group; BB12, *B. animalis* BB12 group. Different lowercase letters in the figure indicate significant differences between groups (*p* < 0.05), *n* = 3.

The concentrations of acetate, butyrate, isobutyrate, and isovalerate in the feces of the mice were lower in the M group than in the NC group, with significantly lower acetate and butyrate levels in the M group (*p* < 0.05; Figure 13A,B). This indicates that following DSS intervention, the microbial balance in the gut was disrupted and the secretion of SCFAs was inhibited. The concentrations of acetate, butyrate, isobutyrate, and isovalerate were higher in the L.pc 63 and BB12 groups than in the M group; among these compounds, acetate and isobutyrate levels were significantly higher in both groups than in the M group (*p* < 0.05; Figure 13A,B). Moreover, the acetate content in the feces was considerably higher in the L.pc 63 group than in the other groups, indicating that *L. paracasei* 63 intervention promoted the secretion of SCFAs in the colon of the mice.

## 4. Discussion

Ulcerative colitis (UC) is characterized by mucosal injury, epithelial barrier dysfunction, excessive inflammation, and, in some cases, systemic immune alterations [2,13]. The dextran sulfate sodium (DSS)-induced colitis model recapitulates key UC-like features, including epithelial damage, diarrhea, hematochezia, weight loss, colon shortening, inflammatory infiltration, and histological injury, and is widely used to evaluate anti-colitis interventions [14]. Probiotics have been reported to alleviate experimental colitis by modulating inflammation, barrier integrity, gut microbiota, and microbial metabolites [15]. In our previous in vitro screening, *L. paracasei* 63 showed anti-inflammatory activity and protective effects on intestinal barrier-related indicators. Based on these findings, the present study further evaluated the protective effects of *L. paracasei* 63 in a DSS-induced mouse model of colitis.

In the present study, DSS administration induced typical UC-like manifestations, including body weight loss, increased DAI, diarrhea, hematochezia, colon shortening, and colonic tissue injury [16]. Oral administration of *L. paracasei* 63 significantly attenuated these pathological changes, indicating its protective effect against DSS-induced colitis. Similar protective effects have been reported for other *L. paracasei* strains, which reduced disease severity, inflammatory infiltration, epithelial damage, and barrier disruption in DSS-induced colitis models [10,17,18]. However, probiotic efficacy is highly strain-dependent; thus, these findings extend previous observations by identifying *L. paracasei* 63 as a candidate strain with anti-colitis potential under the present experimental conditions.

Inflammatory activation is a central feature of DSS-induced colitis and contributes to mucosal injury and disease progression [18]. In this study, *L. paracasei* 63 reduced colonic inflammatory injury and decreased inflammatory cytokine levels. At the molecular level, *L. paracasei* 63 administration was associated with lower TLR4 expression and reduced NF-κB p65 phosphorylation in colon tissues. This is consistent with previous studies showing that TLR4/NF-κB activation promotes pro-inflammatory mediator production and aggravates intestinal inflammation in DSS-induced colitis, whereas probiotic interventions can suppress excessive NF-κB-related inflammatory responses [7,11]. Therefore, reduced TLR4/NF-κB activation may partly explain the anti-inflammatory effects of *L. paracasei* 63, although direct causality requires further validation.

Intestinal epithelial barrier disruption is a key pathological feature of UC and DSS-induced colitis [19]. In this study, DSS exposure increased serum LPS, DAO, and D-LA levels, indicating impaired barrier function and increased intestinal permeability [20]. *L. paracasei* 63 reduced these permeability-related indicators and increased the expression of tight junction proteins, including claudin-1, ZO-1, and occludin. Moreover, *L. paracasei* 63 decreased MLCK and p-MLC2-related changes, which are involved in cytoskeletal contraction and tight junction disruption [21]. These results are consistent with previous studies showing that probiotics can preserve epithelial barrier integrity by restoring tight junction proteins, reducing permeability, and limiting inflammation-associated epithelial injury [22]. Thus, barrier protection may represent an important component of the anti-colitis effects of *L. paracasei* 63.

Gut microbiota dysbiosis and altered microbial metabolites are closely linked to intestinal inflammation and barrier dysfunction in DSS-induced colitis [23]. In this study, *L. paracasei* 63 intervention was associated with changes in gut microbiota composition and increased SCFA levels, particularly acetate. Similar probiotic-mediated increases in SCFAs have been reported in experimental colitis and are often accompanied by improved epithelial barrier function and reduced inflammatory responses [24]. SCFAs can support intestinal homeostasis by serving as energy substrates, strengthening tight junction integrity, regulating immune-cell activity, and limiting excessive cytokine production [25]. Acetate has also been implicated in epithelial protection and immune regulation [26]. However, whether acetate directly mediated the protective effects of *L. paracasei* 63 remains unverified and requires further validation using acetate supplementation, inhibition, or microbiota-targeted interventions.

Beyond local colonic protection, *L. paracasei* 63 reduced serum IL-1β, IL-6, and TNF-α levels and increased IL-10, indicating that colitis improvement was accompanied by attenuated systemic inflammation [27]. This systemic anti-inflammatory pattern is consistent with previous DSS-induced colitis studies showing that probiotic interventions can reduce circulating pro-inflammatory cytokines and promote anti-inflammatory responses [28]. The thymus index was also higher in the *L. paracasei* 63 group than in the DSS model group. Because intestinal inflammation may affect peripheral immune homeostasis and immune-organ status, this finding may suggest an association between intestinal recovery and systemic immune modulation [29]. However, thymus index alone cannot confirm thymic functional recovery or define the underlying immune mechanism. Therefore, the microbiota–acetate–systemic inflammation–thymus relationship should be interpreted as associative rather than causal and requires validation using microbiota depletion, fecal microbiota transplantation, acetate intervention, and immune-cell profiling.

*B. animalis* BB12 was included in this study as a probiotic comparator because it is a well-characterized and widely used commercial probiotic strain [30]. In our study, BB12 also alleviated DSS-induced colitis symptoms, reduced local colonic inflammation, improved intestinal barrier-related indicators, and modulated gut microbiota composition. However, compared with the BB12 group, the *L. paracasei* 63 group showed lower serum IL-1β and TNF-α levels and a higher thymus index. These findings suggest that, under the present experimental conditions, *L. paracasei* 63 showed more pronounced improvements in systemic inflammatory indicators and thymus index than *B. animalis* BB12.

Although the present study provides evidence that *L. paracasei* 63 alleviates DSS-induced colitis and that these effects are accompanied by changes in gut microbiota composition, SCFA production, systemic inflammatory markers, and thymus index, several aspects should be further explored. In particular, the observed association among gut microbiota, SCFAs, and thymus-related changes should be interpreted as hypothesis-generating rather than as direct evidence of a causal microbiota–SCFA–thymus axis. Future studies using approaches such as acetate supplementation, microbiota depletion, fecal microbiota transplantation, or immune-cell-focused analyses would help clarify this relationship. In addition, BB12 was included as a probiotic comparator in this study; however, comparison with a standard therapeutic agent such as mesalazine, as well as inclusion of a healthy probiotic-treated group, would further strengthen the translational interpretation of the findings. Finally, the histological results in this study were mainly used to provide representative morphological evidence of colonic injury and recovery. Further quantitative histopathological assessment may provide additional support for evaluating tissue-level protection.

Although the present study provides evidence that *L. paracasei* 63 alleviates DSS-induced colitis and that these effects are accompanied by changes in gut microbiota composition, SCFA production, systemic inflammatory markers, and thymus index, several limitations should be acknowledged. First, the DSS-induced colitis model is a chemically induced epithelial injury model that reproduces several UC-like pathological features, but it cannot fully recapitulate the chronic, relapsing, multifactorial, and immune-complex nature of human UC [31]. Therefore, the protective effects of *L. paracasei* 63 observed in this model should be further validated in additional experimental models and, ultimately, in clinical studies. Second, although reduced TLR4 expression, decreased NF-κB p65 phosphorylation, improved tight junction protein expression, and altered MLCK/p-MLC2-related changes were observed, these findings remain associative. The present study did not use pathway inhibitors, gene-silencing approaches, or receptor-blocking strategies to directly verify the causal involvement of these signaling pathways. Third, the microbiota- and SCFA-related findings should be interpreted cautiously. The observed changes in microbial composition and acetate levels suggest a potential contribution of the gut microenvironment, but causality was not established by microbiota depletion, fecal microbiota transplantation, germ-free models, acetate supplementation, or acetate inhibition. Similarly, the increased thymus index cannot by itself demonstrate thymic functional recovery or prove a microbiota–SCFA–thymus axis [32]. Fourth, the translational relevance of the findings remains limited because BB12 was used as a probiotic comparator, whereas a standard therapeutic agent such as mesalazine and a healthy probiotic-treated group were not included. In addition, the histological analysis mainly provided representative morphological evidence, and future studies should include quantitative histopathological scoring, immune-cell profiling, dose–response evaluation, and longer-term intervention designs to better define the therapeutic potential and mechanism of *L. paracasei* 63.

Taken together, the present study showed that *L. paracasei* 63 alleviated DSS-induced colitis symptoms, reduced intestinal inflammatory responses, improved intestinal barrier integrity, modulated gut microbiota composition, and increased SCFA levels, particularly acetate. These direct findings were accompanied by reduced systemic inflammatory cytokine levels and an increased thymus index. Based on these observations, the protective effects of *L. paracasei* 63 may be related to multiple coordinated changes involving intestinal barrier function, inflammatory status, gut microbiota composition, microbial metabolites, and systemic immune-related indicators. However, the proposed relationships among these factors remain interpretative and require further mechanistic validation.

## 5. Conclusions

In this study, we systematically evaluated the protective effects of *L. paracasei* 63 against DSS-induced ulcerative colitis in mice. Our findings showed that *L. paracasei* 63 alleviated local colonic inflammation, improved intestinal barrier integrity, and modulated gut microbiota composition and SCFA production. These intestinal changes were accompanied by reduced systemic inflammation and an increased thymus index. Collectively, these results suggest that the protective effects of *L. paracasei* 63 are associated with improvements in intestinal barrier integrity, gut microbiota composition, SCFA production, and systemic immune-related indicators, highlighting its potential as a promising probiotic candidate for the management of ulcerative colitis.

## Figures and Tables

**Table 1:** A list of real-time quantitative PCR primers used in this study.

Gene	Forward Primer (5′ → 3′)	Reverse Primer (5′ → 3′)
IL-1β	CTCCACCTCCAGGGACAGGATATG	TCATCTTTCAACACGCAGGACAGG
IL-6	GGTGTTGCCTGCTGCCTTCC	GTTCTGAAGAGGTGAGTGGCTGTC
IL-10	AGGAGGTGATGCCCCAAGCTGA	TCGATGACAGCGCCGTAGCCT
TNFα	TATTGCCGAAATGTGCTCAAGGGC	GAGTAGACAAGGTACAACCC
β-actin	GTGGACCTGACCTGCCGTCTAG	GAGTGGGTGTCGCTGTTGAAGTC

## Data Availability

The original contributions presented in this study are included in the article. The 16S rRNA gene sequencing data generated and analyzed in this study, including the raw sequencing data and processed datasets, are available from the corresponding author upon reasonable request. Other data supporting the findings of this work are also available from the corresponding author upon reasonable request.

## Abbreviations

The following abbreviations are used in this manuscript:

UC	Ulcerative colitis
SCFAs	short-chain fatty acids
ELISA	Enzyme-linked immunosorbent assay
qRT-PCR	Quantitative real-time polymerase chain reaction
